# Supplementary material for: Praziquantel inhibits Caenorhabditis elegans development and species-wide differences might be cct-8-dependent
Source: PLoS One. 2023 Aug 10;18(8):e0286473. doi: 10.1371/journal.pone.0286473 (PMC10414639; doi:10.1371/journal.pone.0286473)
Supplement: S1 Table — (PDF) [file pone.0286473.s001.pdf]

S1 Table
Primer sequences for qPCR

Gene	Forward	Reverse	Annealing temp ˚C
rrn-1.1 (18S rRNA)	TTGCGTCAACTGTGGTCGTG	CCAACAAAAAGAACCGAAGTCCTG	58
hsp-16.2	ACTTTACCACTATTTCCGTCCAGC	CCTTGAACCGCTTCTTTCTTTG	58/60
cct-8	TGTCGTCGCCGCTGGAAAGTTC	AAGAGTTTCGAGTGCGTGGGCG	60
rpl-26	ACTTCAACGCTCCATCCCAC	AACGACGACCTCATCATCGG	60
hsp-70	GTACTACGTACTCATGTGTCGGTATTTATC	ACGGGCTTTCCTTGTTTTCC	60
