# Supplementary material for: Praziquantel inhibits Caenorhabditis elegans development and species-wide differences might be cct-8-dependent
Source: PLoS One. 2023 Aug 10;18(8):e0286473. doi: 10.1371/journal.pone.0286473 (PMC10414639; doi:10.1371/journal.pone.0286473)
Supplement: S2 Table — (PDF) [file pone.0286473.s002.pdf]

S2 Table

Oligonucleotide sequences for generation of ECA485 *cct-8*(*ean8*) in an N2 background and ECA601 *cct-8*(*ean39*) in a JU775 background.

**Component**	**Sequence**
N2 gRNA	GGTCTTTAAACGTGGAGCCG
N2 to JU775 allele repair template	TTCAACGTTGATAATATCAGAATCTGCAAGATTATTGGCTCAGGAGTTCATAC TTCAACAGTTATGAACGGAATGGTCTTTAAACGTGTAGCCGAAGGAGAAATT CGTGAAGCTCGCGACGCTAGAATTGCA
JU775 gRNA	GGTCTTTAAACGTGTAGCCG
JU775 to N2 repair template	TGCAATTCTAGCGTCGCGAGCTTCACGAATTTCTCCTTCGGCTCCACGTTTA AAGACCATTCCGTTCATAACTGTTGAAGTATGAACTCCTGAGCCAATAATCTT GCAGATTCTGATATTATCAACGTTGAA
oECA1155 genotyping forward primer	CCGTCAATATATTCGTTCAGCAATCACCTC
oECA1157 genotyping reverse primer	GGAGAAGTTCCTAAGCTCATCAGCATTC
