# Supplementary material for: Praziquantel inhibits Caenorhabditis elegans development and species-wide differences might be cct-8-dependent
Source: PLoS One. 2023 Aug 10;18(8):e0286473. doi: 10.1371/journal.pone.0286473 (PMC10414639; doi:10.1371/journal.pone.0286473)
Supplement: S3 Table — (PDF) [file pone.0286473.s003.pdf]

S3 Table

Two-way ANOVA results for dose response assay of racemate praziquantel (PZQ), (*S*)-PZQ, and (*R*)-PZQ. Significance: *** p< 0.001, ** p< 0.01, * p< 0.05.

A	Dose (mM)	Effect	DFn	DFd	F	p
	0	drug	2	131	0	0
		strain	1	131	0	0
		drug:strain	2	131	0	0
	250	drug	2	134	1.289	$2.79\ 10^{-1}$
		strain	1	134	31.745	0 ***
		drug:strain	2	134	0.225	$7.99\ 10^{-1}$
	500	drug	2	132	2.012	$1.38\ 10^{-1}$
		strain	1	132	52.439	0 ***
		drug:strain	2	132	1.501	$2.27\ 10^{-1}$
	1000	drug	2	135	0.551	$5.78\ 10^{-1}$
		strain	1	135	194.236	0 ***
		drug:strain	2	135	2.752	$6.7\ 10^{-2}$
	1500	drug	2	133	2.329	$1.01\ 10^{-1}$
		strain	1	133	202.207	0 ***
		drug:strain	2	133	8.121	$4.70\ 10^{-4}$ ***
	2000	drug	2	133	1.688	$1.89\ 10^{-1}$
		strain	1	133	172.858	0 ***
		drug:strain	2	133	14.179	$2.62\ 10^{-6}$ ***
	3000	drug	2	131	3.319	$3.9\ 10^{-2}$ *
		strain	1	131	126.732	0 ***
		drug:strain	2	131	6.239	$3.0\ 10^{-3}$ ***

B	Strain	Dose	Effect	DFn	DFd	F	p
	N2	1500	drug	2	67	8.797	$4.05\ 10^{-4}$ ***
	JU775		drug	2	66	1.768	$1.79\ 10^{-1}$
	N2	2000	drug	2	68	16.953	$1.06\ 10^{-6}$ ***
	JU775		drug	2	65	2.487	$9.1\ 10^{-2}$
	N2	3000	drug	2	68	8.411	$5.45\ 10^{-4}$ ***
	JU775		drug	2	63	3.253	$4.5\ 10^{-2}$ *
