# Supplementary material for: Praziquantel inhibits Caenorhabditis elegans development and species-wide differences might be cct-8-dependent
Source: PLoS One. 2023 Aug 10;18(8):e0286473. doi: 10.1371/journal.pone.0286473 (PMC10414639; doi:10.1371/journal.pone.0286473)
Supplement: S4 Table — (PDF) [file pone.0286473.s004.pdf]

S4 Table

post hoc Tukey HSD Test for the effect of drug types (racemate praziquantel (PZQ), (*S*)-PZQ, and (*R*)-PZQ) on development at doses 1500, 2000, and 3000 mM. Significance: *** p< 0.001, ** p< 0.01, * p< 0.05.

Strain	Dose	Term	Group 1	Group 2	Estimate	conf-low	conf.high	p.adj
N2	1500	drug	PZQ	(*S*)-PZQ	-0.199	-0.323	-0.075	$7.79\ 10^{-4}$ ***
		drug	PZQ	(*R*)-PZQ	-0.173	-0.297	-0.048	$3.86\ 10^{-3}$ **
		drug	(*S*)-PZQ	(*R*)-PZQ	0.026	-0.099	0.151	$8.71\ 10^{-1}$
JU775		drug	PZQ	(*S*)-PZQ	0.041	-0.087	0.168	$7.27\ 10^{-1}$
		drug	PZQ	(*R*)-PZQ	0.099	-0.028	0.224	$1.55\ 10^{-1}$
		drug	(*S*)-PZQ	(*R*)-PZQ	0.058	-0.071	0.187	$5.31\ 10^{-1}$
N2	2000	drug	PZQ	(*S*)-PZQ	-0.155	-0.234	-0.076	$3.41\ 10^{-5}$ ***
		drug	PZQ	(*R*)-PZQ	-0.172	-0.249	-0.093	$4.22\ 10^{-6}$ ***
		drug	(*S*)-PZQ	(*R*)-PZQ	-0.016	-0.095	0.062	$8.73\ 10^{-1}$
JU775		drug	PZQ	(*S*)-PZQ	0.083	-0.021	0.187	$1.45\ 10^{-1}$
		drug	PZQ	(*R*)-PZQ	0.082	-0.020	0.183	$1.39\ 10^{-1}$
		drug	(*S*)-PZQ	(*R*)-PZQ	-0.001	-0.106	0.104	1.00
N2	3000	drug	PZQ	(*S*)-PZQ	-0.124	-0.218	-0.029	$7.01\ 10^{-3}$ **
		drug	PZQ	(*R*)-PZQ	-0.150	-0.244	-0.056	$7.69\ 10^{-4}$ ***
		drug	(*S*)-PZQ	(*R*)-PZQ	-0.026	-0.121	0.068	$7.83\ 10^{-1}$
JU775		drug	PZQ	PZQ_p1	-0.070	-0.227	0.088	$5.43\ 10^{-1}$
		drug	PZQ	PZQ_p2	0.100	-0.054	0.253	$2.71\ 10^{-1}$
		drug	PZQ_p1	PZQ_p2	0.169	0.009	0.330	$3.68\ 10^{-2}$ *
