# Supplementary material for: Praziquantel inhibits Caenorhabditis elegans development and species-wide differences might be cct-8-dependent
Source: PLoS One. 2023 Aug 10;18(8):e0286473. doi: 10.1371/journal.pone.0286473 (PMC10414639; doi:10.1371/journal.pone.0286473)
Supplement: S5 Table — (PDF) [file pone.0286473.s005.pdf]

S5 Table

Genes found in the fine mapped region on chromosome IV position 939,925 to 1,334,212 in order of descending significance.

Gene	WormBase Gene ID	-log₁₀(*p*)	Position	Strand
znf-782	WBGene00021931	15.2719313	1058076 - 1067525	-
Y104H12D.4.1	WBGene00022428	14.6938193	1182592 - 1186625	+
Y104H12D.2.1	WBGene00022426	14.550864	1178022 - 1182864	+
cct-8	WBGene00021934	13.8475421	1087108 - 1093920	-
cox-18	WBGene00021932	13.8475421	1097453 - 1102980	+
C44B12.3.1	WBGene00016637	13.4569554	1104766 - 1105130	+
ost-1	WBGene00003893	13.4569554	1107396 - 1110872	+
srx-13	WBGene00005904	13.4569554	1114697 - 1118666	-
C44B12.6a.1	WBGene00016639	13.4569554	1128841 - 1132683	-
cec-2	WBGene00016796	13.4569554	1170708 - 1177776	+
C50A2.7.1	WBGene00219437	13.1404204	1163426 - 1164746	+
ets-8	WBGene00016798	11.9162309	1159361 - 1160486	-
dcap-1	WBGene00021929	11.6314545	1052524 - 1054794	-
mrps-25	WBGene00021920	11.6314545	1056892 - 1057619	+
csn-4	WBGene00000816	11.6314545	1077463 - 1087013	-
spl-3	WBGene00022427	8.72164993	1193065 - 1201901	+
perm-2	WBGene00016636	6.98647278	1126790 - 1128865	+
clec-165	WBGene00009523	6.98606174	1245171 - 1246886	-
clec-166	WBGene00009518	6.18200539	1249192 - 1251342	-
F53H1.4a.1	WBGene00018778	5.85412641	1310166 - 1328243	+
F53H1.3a.1	WBGene00018777	5.85412641	1329162 - 1339376	+
plx-1	WBGene00004047	5.47501397	959521 - 984395	+
clec-167	WBGene00009517	5.33592578	1253137 - 1255732	-
dsl-2	WBGene00001104	4.78893438	1202651 - 1204970	-
clec-168	WBGene00009520	4.68194864	1256969 - 1259344	+
lep-2	WBGene00002278	4.65543033	992283 - 996148	+
Y55F3AM.9a.1	WBGene00021926	4.65543033	1006083 - 1009734	-
rbm-39	WBGene00021921	4.65543033	1012624 - 1019558	+
dsl-1	WBGene00001103	4.47344918	1207218 - 1208080	-
Y55F3AM.11.1	WBGene00021928	4.39869275	1038667 - 1044387	-
F38A1.11.1	WBGene00009524	3.92878775	1247386 - 1249025	+

F38A1.9.1	WBGene00009522	3.89471368	1244389 - 1245344	-
W09G12.10.1	WBGene00044463	3.74266005	1219338 - 1221287	-
plst-1	WBGene00022425	3.72350817	1289627 - 1296889	+
Y55F3AR.2.1	WBGene00021933	3.63343718	1094541 - 1097342	+
clec-170	WBGene00009515	3.41480555	1266866 - 1274425	+
W09G12.9.1	WBGene00021123	3.37620238	1216590 - 1218527	+
F38A1.6.1	WBGene00009519	3.09693699	1251861 - 1252387	+
Y55F3AM.13.1	WBGene00021930	2.99832162	1055023 - 1056313	-
srx-14	WBGene00005905	2.93792007	1111462 - 1113738	-
F38A1.15.1	WBGene00044908	2.80050765	1241332 - 1243395	-
mdt-20	WBGene00007020	2.65862452	1187802 - 1192978	+
F38A1.8.1	WBGene00009521	2.50642859	1232825 - 1239852	-
W09G12.7.1	WBGene00021121	2.18579811	1229080 - 1230793	+
egrh-2	WBGene00043705	2.14868016	984384 - 990452	-
atg-3	WBGene00021922	2.12753155	1010512 - 1012502	+
Y55F3AM.21a.2	WBGene00219399	2.0318254	1049522 - 1051664	-
C09E9.1d.1	WBGene00007476	2.02773753	1274868 - 1277062	+
W09G12.8.1	WBGene00021122	1.82396246	1226182 - 1227668	+
W09G12.6.1	WBGene00021120	1.82396246	1231889 - 1232866	+
srx-12	WBGene00005903	1.44788895	1045983 - 1049506	+
immp-2	WBGene00021925	1.31260533	1003595 - 1005861	-
clec-169	WBGene00009526	1.23199512	1261324 - 1264622	+
ddx-46	WBGene00018776	1.02494277	1297222 - 1309650	-
Y55F3AM.5.1	WBGene00021923	1.00364762	999214 - 1003626	+
faah-3	WBGene00019068	0.72927396	940017 - 945653	-
perm-4	WBGene00016638	0.69960197	1121494 - 1125107	-
F38A1.13.1	WBGene00009525	0.69960197	1265228 - 1265848	+
