# Supplementary material for: Praziquantel inhibits Caenorhabditis elegans development and species-wide differences might be cct-8-dependent
Source: PLoS One. 2023 Aug 10;18(8):e0286473. doi: 10.1371/journal.pone.0286473 (PMC10414639; doi:10.1371/journal.pone.0286473)
Supplement: S6 Table — (PDF) [file pone.0286473.s006.pdf]

S6 Table

Two-way-ANOVA results for *cct-8*, *hsp-16.2*, and *hsp-70* expression in the strains N2 and JU775 in control (1% DMSO) and PZQ conditions (1 mM PZQ in 1% DMSO). Significance: *** p< 0.001, ** p< 0.01, * p< 0.05.

Gene	Descriptor	Df	Sum Sq	Mean Sq	F value	*p* value
cct-8	Strain	1	7.111	7.111	22.610	$2.7\ 10^{-5}$ ***
	Condition	1	0.176	0.176	0.559	0.45905
	Plate	1	3.793	3.793	12.061	$1.28\ 10^{-3}$ **
	Strain:Condition	1	0.241	0.241	0.766	0.38684
	Residuals	39	12.266	0.315		
***cct-8* Plate 1**†	Strain	1	4.573	4.573	13.445	$1.91\ 10^{-3}$ **
	Condition	1	0.011	0.011	0.031	0.86190
	Strain:Condition	1	1.301	1.301	3.825	0.06714
	Residuals	17	5.783	0.340		
***cct-8* Plate 2**†	Strain	1	2.310	2.3102	9.760	$5.59\ 10^{-3}$ **
	Condition	1	0.693	0.6930	2.928	0.10336
	Strain:Condition	1	0.234	0.2337	0.987	0.33294
	Residuals	19	4.497	0.2367		
hsp-16.2	Strain	1	3.72	3.724	0.615	0.437
	Condition	1	3.49	3.491	0.576	0.452
	Plate	1	0.13	0.130	0.021	0.884
	Strain:Condition	1	15.90	15.905	2.626	0.113
	Residuals	42	254.38	6.057		
hsp-70	Strain	1	0.5656	0.5656	18.560	$1.04\ 10^{-4}$***
	Condition	1	0.0307	0.0307	1.007	0.321628
	Plate	1	0.0133	0.0133	0.437	0.512301
	Strain:Condition	1	0.0304	0.0304	0.997	0.324063
	Residuals	40	1.2190	0.0305		

† For *cct-8*, the qPCR plate had an effect on the results. Therefore, plates 1 and 2 were analyzed individually.
