# Supplementary material for: Praziquantel inhibits Caenorhabditis elegans development and species-wide differences might be cct-8-dependent
Source: PLoS One. 2023 Aug 10;18(8):e0286473. doi: 10.1371/journal.pone.0286473 (PMC10414639; doi:10.1371/journal.pone.0286473)
Supplement: S1 Fig — Peak 1 = (R)-PZQ, peak 2 = (S)-PZQ. (PDF) [file pone.0286473.s007.pdf]

Final Analysis and Purification Report

Requestor: Eric Andersen
Company: Northwestern University

Vial w/o cap + sample: 5.999 g
Vial w/o cap: 5.491 g

Sample ID: Praziquantel
Date received: 11-11-2019
Date shipped: 12-3-2019
Amount stated: 500 mg
Amount transf.: 508 mg

**Analysis Summary:**

The following SFC separation (conditions listed below) yielded 276 mg of peak-1 and 283 mg of peak-2. Waste is available upon request. Chromatograms are included in this report.

Preparative Method:

IA (2 x 15 cm)
40% ethanol (0.1% DEA)/$CO_2$, 100 bar
65 mL/min, 220 nm
inj vol.: 0.5 mL, 30 mg/mL ethanol:DCM

Analytical Method:

IA (25 x 0.46 cm)
50% ethanol (DEA)/$CO_2$, 120 bar
3 mL/min, 220, 254 and 280 nm

Sample: Praziquantel

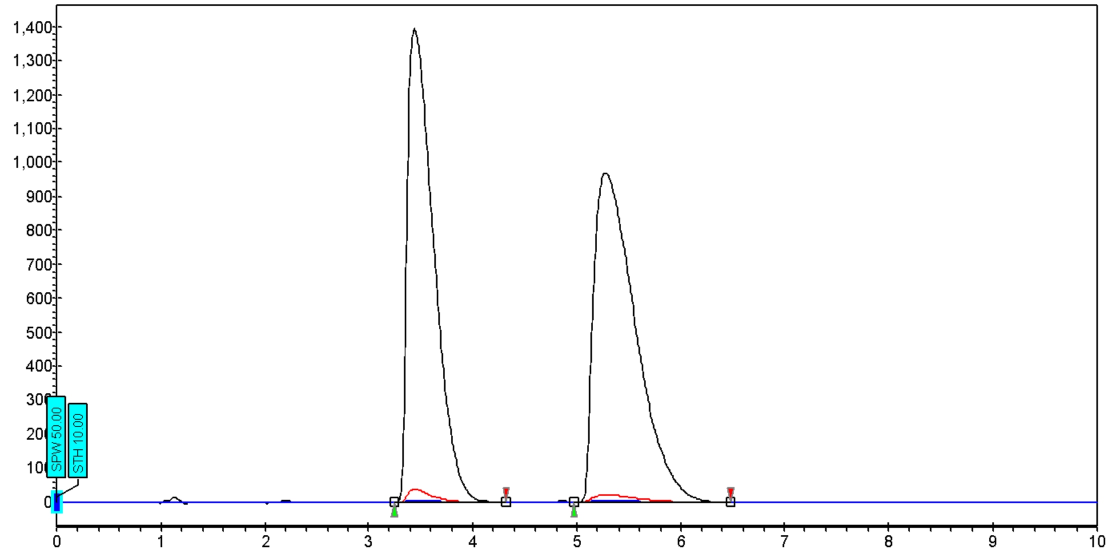

Lotus Separations

Final Analysis and Purification Report

Sample: Praziquantel peak-1

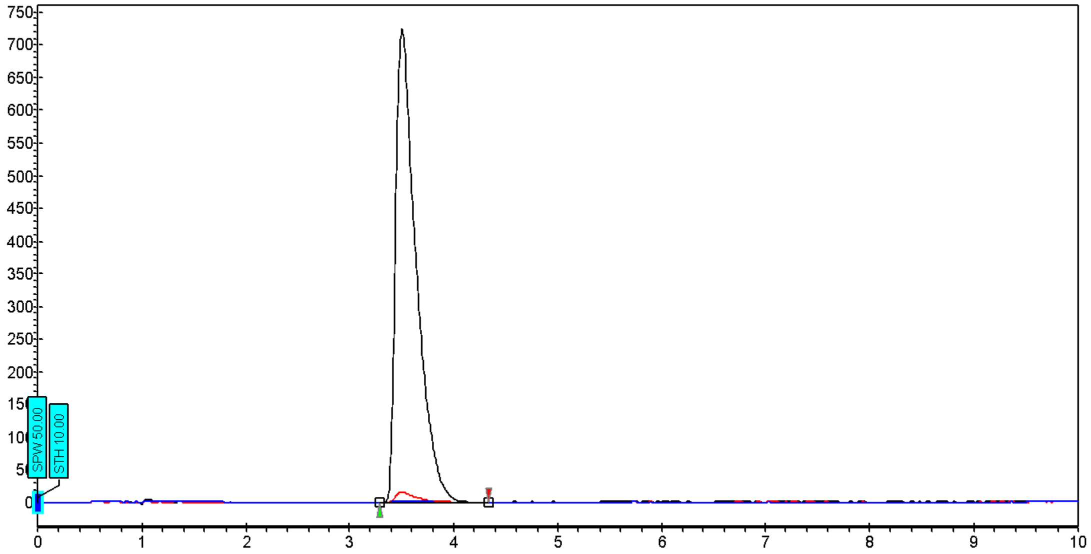


| Index | Time (min) | Area (%) 220 nm |
|---|---|---|
| Peak-1 | 3.50 | 100.00 |
| Total | | 100.00 |

Sample: Praziquantel peak-2

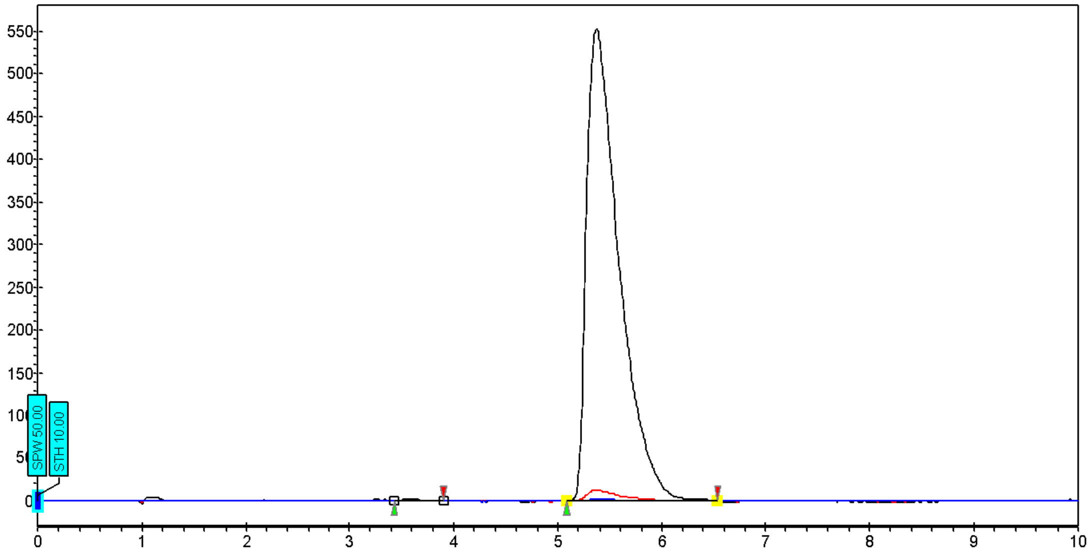


| Index | Time (min) | Area (%) 220 nm |
|---|---|---|
| Peak-1 | 3.61 | 0.181 |
| Peak-2 | 5.37 | 99.819 |
| Total | | 100.00 |
