# Supplementary material for: Praziquantel inhibits Caenorhabditis elegans development and species-wide differences might be cct-8-dependent
Source: PLoS One. 2023 Aug 10;18(8):e0286473. doi: 10.1371/journal.pone.0286473 (PMC10414639; doi:10.1371/journal.pone.0286473)
Supplement: S2 Fig — (PDF) [file pone.0286473.s008.pdf]

S2 Fig

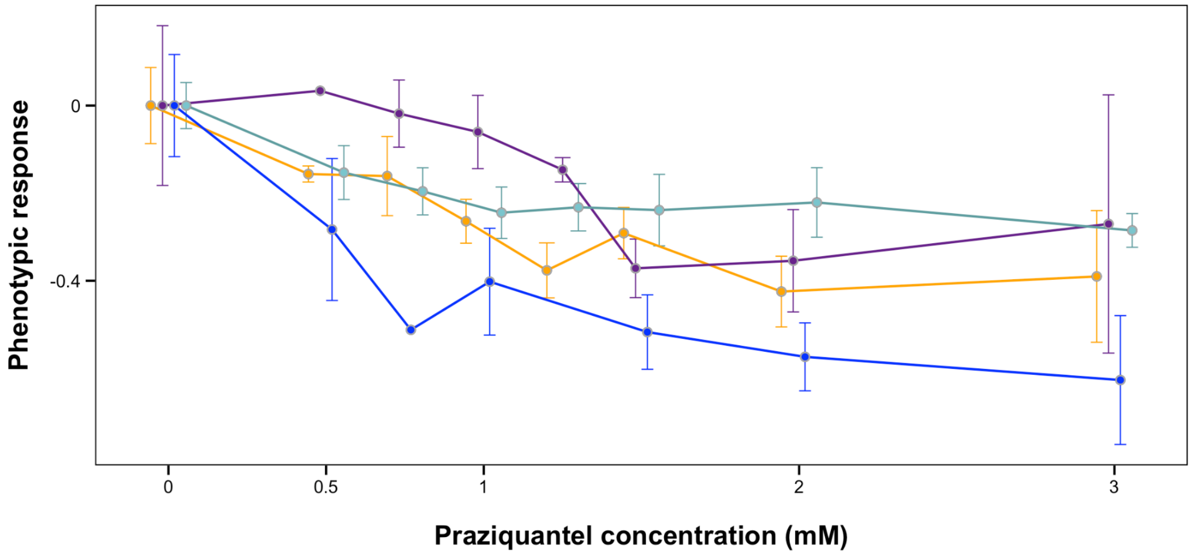

The effect of increasing concentrations of Praziquantel (PZQ) on *C. elegans* development. Dose responses for the N2 (orange), JU775 (purple), DL238 (lightblue), and CB4856 (blue) strains in the PZQ racemate. The phenotypic responses are median optical density normalized by animal length.
